# How the Realism of Robot Is Needed for Individuals With Autism Spectrum Disorders in an Interview Setting

**DOI:** 10.3389/fpsyt.2019.00486

**Published:** 2019-07-11

**Authors:** Hirokazu Kumazaki, Taro Muramatsu, Yuichiro Yoshikawa, Yoshio Matsumoto, Masutomo Miyao, Hiroshi Ishiguro, Masaru Mimura, Yoshio Minabe, Mitsuru Kikuchi

**Affiliations:** ^1^Department of Clinical Research on Social Recognition and Memory, Research Center for Child Mental Development, Kanazawa University, Ishikawa, Japan; ^2^National Center of Neurology and Psychiatry, Department of Preventive Intervention for Psychiatric Disorders, National Institute of Mental Health, Tokyo, Japan; ^3^Department of Neuropsychiatry, Keio University School of Medicine, Tokyo, Japan; ^4^Department of Systems Innovation, Graduate School of Engineering Science, Osaka University, Toyonaka, Japan; ^5^JST ERATO ISHIGURO Symbiotic Human-Robot Interaction, Toyonaka, Japan; ^6^Service Robotics Research Group, Intelligent Systems Institute, National Institute of Advanced Industrial Science and Technology, Ibaraki, Japan; ^7^Department of Psychosocial Medicine, National Center for Child Health and Development, Tokyo, Japan

**Keywords:** autism spectrum disorders, android robot, appearance, interview, motivation, humanness

## Abstract

The preliminary efficacy of interview training using an android robot whose appearance and movements resemble those of an actual human for treating social and communication difficulties in individuals with autism spectrum disorders (ASD) has been demonstrated. Patient preferences regarding the appearance of robots are crucial for incentivizing them to undergo robot-assisted therapy. However, very little is known about how the realistic nature of an android robot is related to incentivizing individuals with ASD in an interview setting. In this study, individuals with ASD underwent an interview with a human interviewer and an android robot. Twenty-three individuals with ASD (age, 17–25 years) participated in this study. After the interview, the participants were evaluated in terms of their motivation to practice an interview with an android robot and their impression of the nature of the android robot in terms of humanness. As expected, subjects exhibited higher motivation to undergo interview training with an android robot than with a human interviewer. Higher motivation to undergo an interview with the android robot was negatively correlated with the participants’ impressions of the extent to which the android robot exhibited humanness. This study brings us one step closer to understanding how such an android robot should be designed and implemented to provide sufficiently realistic interview training that can be of therapeutic value.

## Introduction

Social and communication difficulties are among the hallmark clinical features of autism spectrum disorders (ASD). The social difficulties associated with ASD could have a strong effect on the quality of interactions with other individuals (1, 2) and could become increasingly debilitating and distressing during adolescence (3). The accumulated intervention literature to date suggests that social communication approaches are effective when individuals with ASD have high motivation (4).

There is a growing body of literature indicating that many individuals with ASD have the motivation and aptitude for using technology (5). An android robot is a robot with an appearance and movements resembling those of an actual human. Given the high technology behind the android robot, it is expected that an android robot may be favored by individuals with ASD.

Our previous study demonstrated the preliminary efficacy of interview training using an android robot for treating social and communication difficulties in individuals with ASD (6). In the study, all participants concentrated during the trials and were highly motivated throughout the experiment. In addition, creating intelligent three-dimensional learning environments using an android robot may contribute to the generalization of social skills obtained in the robot sessions to subsequent interactions with humans. Given that the ultimate goal of robot-assisted ASD therapy is the generalization of social skills obtained in the robot sessions, robots that are more human-like may be advantageous.

Human beings have a unique sense of humanness, a special sense of “human nature” that involves emotion, warmth, and cognitive flexibility, as opposed to “mechanistic” dehumanization (7). The concept of the uncanny valley is the proposed relationship between the humanness of an entity and the perceiver’s affinity for it and suggests that android robots that appear almost, but not exactly, like real human beings elicit uncanny, or strangely familiar, feelings of eeriness and revulsion in observers. That is, an android robot with an appearance resembling humans that is not an exact replica of humans can promote repulsion and a sensation of eeriness. In addition, considering that preference for the appearance of a robot is suggested to vary tremendously across individuals with ASD (8), pursuing optimal “humanness” for an android robot is an important subject in designing the proper interview training. The question remains as to how the impression of the humanness of an android robot relates to motivation in an interview setting using an android robot for individuals with ASD.

We investigated the relationship between the impression of the humanness of an android robot and the motivation of individuals with ASD with respect to an interview setting using an android robot. A greater understanding of the relationship could provide insight into developing a future therapeutic android robot to manage social communication difficulties in an interview setting for individuals with ASD.

## Methods

### Participants

The study was approved by the Ethics Committee of Kanazawa University and was conducted in accordance with the Declaration of Helsinki. Participants were recruited from our medical center and related clinic specialized in developmental disorders and related conditions. After a complete explanation of the study, all participants provided written, informed consent. All participants and their guardians agreed to participate in the study. The inclusion criteria for participants were 1) diagnosis of ASD based on the *Diagnostic and Statistical Manual of Mental Disorders* (DSM-5) (9), 2) aged 17–25 years, and 3) IQ ≥ 70. The exclusion criteria for the ASD group were medical conditions associated with ASD (e.g., fragile X mental retardation 1, Rett syndrome, Shank3). To exclude other psychiatric diagnoses, the Mini-International Neuropsychiatric Interview (10) was administered. The participants were diagnosed by a psychiatrist with >10 years of experience working with ASD patients using the criteria in the DSM-5 and standardized criteria taken from the Diagnostic Interview for Social and Communication Disorders (DISCO) (11) at the time of enrolment in the study. The DISCO is reported to have good psychometric properties (12). All participants who were diagnosed with childhood autism or Asperger’s syndrome with DISCO were included in this study. IQ eligibility was confirmed using the Wechsler Adult Intelligence Scale—Fourth Edition.

### Procedure

**Figure 1:**
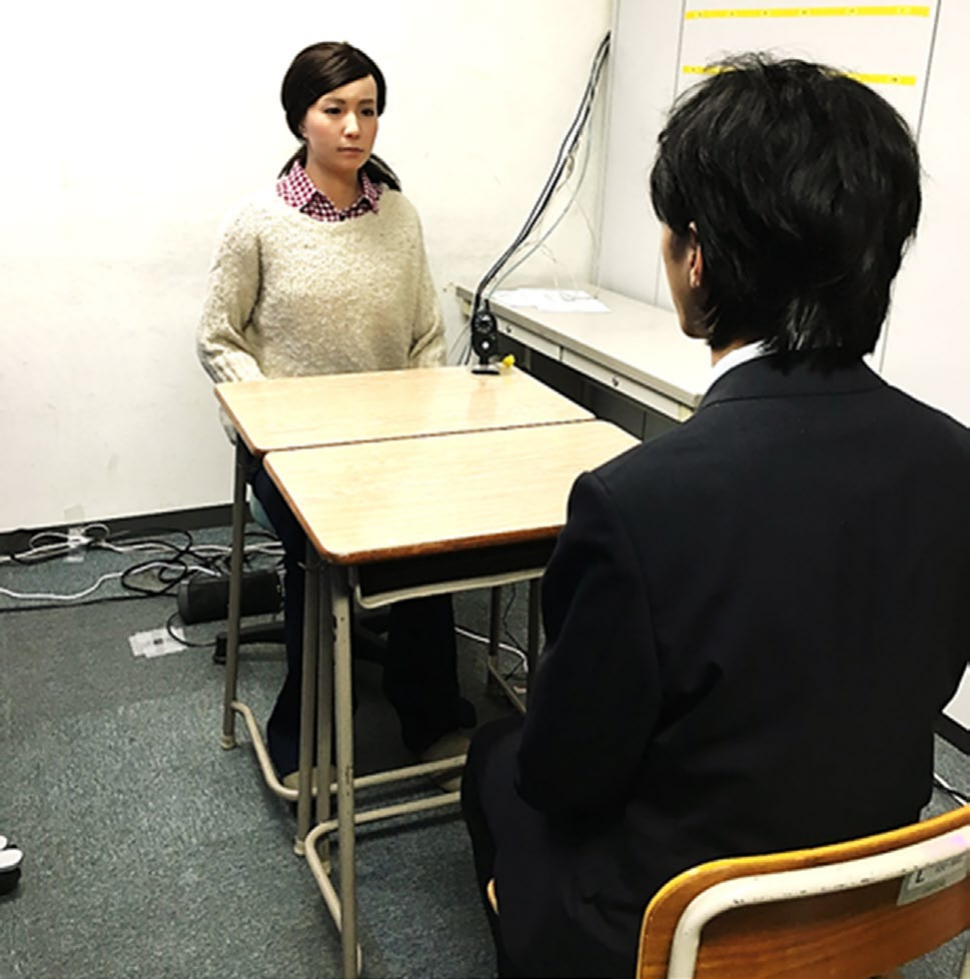
Example of how participants typically interacted with the android.

Prior to each trial, the android robot and a human interviewer were located in different areas; each was seated at their desk. To elicit the belief that the android robot was behaving and responding autonomously, we adopted a remote control system similar to those conventionally used in robotics research (13). The android robot was operated by researchers who were sat in front of a computer terminal located against the wall in the experimental room in order that the researchers were not visible to the participant during the trial. The researcher could monitor the interlocutor *via* video. Participants were brought to the interview room individually by their caregivers, who were present throughout the procedure, but were not within the view of the participants. They remained in the same room in case participants were confused about what they needed to do. The participant sat in front of a human interviewer or the android robot. **Figure 1** provides an example of how participants typically interacted with the android robot. An interviewer or the android robot remotely controlled by an operator conducted an interview according to a script that was prepared in advance. The content of the interview is described elsewhere (see **Supplementary Material**). The scripts were semi-structured to allow interviewers to control the level of difficulty and content of the conversation. Interviewers performed the same sequence of reactions according to a guide for reaction sequences. To reduce sequence effects, the interview orders were counterbalanced between two groups. Participants in the first group (*n* = 12) first underwent an interview with a human, followed by an interview with the android robot. Participants in the second group (*n* = 11) underwent an interview with the android robot first and an interview with a human second. The average duration of each interview was approximately 10 min.

### Android Robot

**Figure 2:**
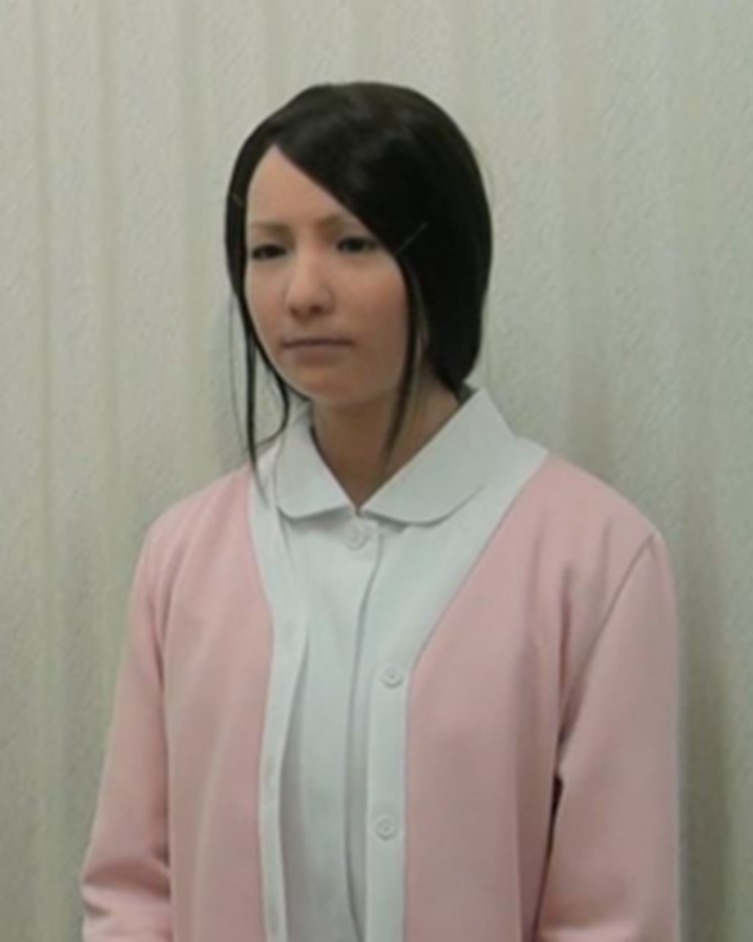
Actroid-F (android).

The android robot used in this study was Actroid-F (**Figure 2**; Kokoro Co. Ltd.), a type of android robot designed with an appearance strongly similar to that of a real female human (6, 8, 14–16). Its artificial body is designed with the proportions, facial features, hair color, texture, and style as a human. It has 12 degrees of freedom (DOF) in total, seven of which are for the face [eye movements (2), mouth movements (1), eyebrows (2), eyelids (1), and smiling (1)]. Blinking, breathing, gaze, and head movements are automatically generated. It can also be operated by a remote conversation system (e.g., teleoperation) and incorporate changes in facial expression (smiling, nodding, and brow movements) during speech. Its face can exhibit a range of expressions, albeit in a less sophisticated manner than a real human face.

### Questionnaires

Prior to the interview, all participants completed the Autism Spectrum Quotient, Japanese version (AQ-J) (17) for the evaluation of ASD-specific behaviors and symptoms. The AQ-J is a short questionnaire with five subscales (social skills, attention switching, attention to detail, imagination, and communication), and results from this measure have been replicated across both culture (17) and age (18, 19). The AQ is also sensitive to the broader autism phenotype (20). The reliability of the AQ-J for both test–retest (γ = 0.87) and inter-rater measures (γ = 0.81) have been observed to be significantly high (21).

After the interview, participants answered two yes/no questions, namely, “Do you want to practice an interview with an android robot again?” and “Do you think that practicing an interview with an android robot makes you proficient?” To evaluate the motivation for interview settings, participants also answered a question that assessed the motivation for an interview with an android robot compared with the motivation for an interview with the human. The item was scored using a five-point Likert scale. The question was “How motivated are you to undergo interview training with an android robot compared to a human?” Participants responded on a scale of −2 to +2 (−2 = motivated to undergo interview training significantly less with an android robot than with a human, −1 = motivated to undergo interview training less with an android robot than with a human, 0 = motivated to undergo interview training with an android robot as much as with a human, 1 = motivated to undergo interview training more with an android robot than with a human, and 2 = motivated to undergo interview training significantly more with an android robot than with a human).

To evaluate the impression of the humanness of the android robot used in the interview setting, we asked questions used in a previous study (7). The participants evaluated their impression of the human nature of the android robot in terms of humanness (1 = *mechanistic*, 5 = *human-like*), emotion (1 = *lacking in emotion*, 5 = *rich in emotion*), animatedness (1 = *inanimate*, 5 = *animate*), naturalness (1 = *unnatural*, 5 = *natural*), familiarity (1 = *unfamiliar*, 5 = *familiar*), warmth (1 = *cold*, 5 = *warm*), complexity (1 = *simple*, 5 = *complex*), and regularity (1 = *random*, 5 = *regular*). A total score was also calculated by summing the scores for these individual items.

### Statistical Analysis

We performed statistical analyses using SPSS version 24.0 (IBM, Armonk, NY, USA). We used Spearman’s rank correlation coefficient to explore the relationships between the impressions of the humanness of the android robot and the motivation of individuals with ASD for interview training using an android robot. We also compared the motivation for interview training with an android robot with the motivation for interview training with a human using the Wilcoxon signed-rank test (against zero). We set the significance level at .05 for all results.

## Results

In total, 23 individuals with ASD took part in the study. The details are presented in **Table 1**. We carefully observed participant performance and confirmed that all participants were concentrating during the trials and were highly motivated from the start to the end of the experiment. All participants completed the experimental procedure and the questionnaires. In response to the question, “Do you want to practice an interview with an android robot again?” 20 participants (87.0%) indicated “yes.” In response to the question, “Do you think practicing an interview with an android robot will make you proficient?” 20 participants (87.0%) indicated “yes.”

**Table 1:** Descriptive statistics of participants (*n* = 23).

Characteristics	M (SD)
Age in years	19.7 (3.1)
Gender (male:female)	17:6
Full-scale IQ	86.4 (11.2)
AQ-J	29.6 (3.8)

M, mean; SD, standard deviation; AQ-J, Autism Spectrum Quotient, Japanese version. In the AQ-J, higher scores reflect a greater number of autism spectrum disorder (ASD)-specific behaviors.

**Table 2** indicates the correlations between the impression of the humanness of the android robot and the motivation to engage in an interview with an android robot. Spearman’s rank correlation coefficients revealed significant negative correlations between the motivation of individuals with ASD to engage in an interview and the impressions of the humanness, emotion, warmth, and the total score of the android robot.

**Table 2:** Correlations between the motivation of individuals with ASD to engage in an interview with an android and the impression of the human nature of the android.

Human nature	*n* = 23
Humanness	−0.53*
Emotion	−0.64**
Animatedness	−0.31
Naturalness	−0.23
Familiarity	−0.17
Warmth	−0.43*
Complexity	−0.29
Regularity	−0.06
Total	−0.64**

*p < .05, **p < .01.

**Figure 3:**
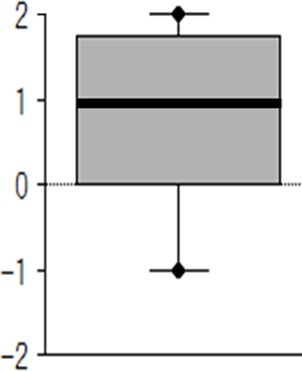
Box plot summarizing the subjective relative motivation for interview setting (android vs. human condition). Positive values denote greater motivation to engage in interview training with an android than with a human. Zero indicates equal motivation for interview training with an android or a human. Negative values denote greater motivation to engage in interview training with a human than an android. The bold horizontal line crossing the box is the median; the bottom and top of the box are the lower and upper quartiles, respectively; and the whiskers are the minimum and maximum values. A Wilcoxon signed-rank test (against zero) demonstrated that the android robot condition was superior to the human condition (*n* = 23, *z* = −2.46, *p* = .014).

With regard to the motivation to engage in interview training, as presented in **Figure 3**, a Wilcoxon signed-rank test (against zero) demonstrated that the android robot condition was superior to the human condition (*n* = 23, *z* = −2.46, *p* = .014).

## Discussion

The most notable finding of this study was that the motivation to undergo interview training using an android robot was negatively correlated with the impressions of the humanness of the android robot. That is, individuals with ASD who reported an impression of reduced humanness of the android robot had higher incentive for interview training using an android robot. Interventions for individuals with ASD require particularly high participant motivation (4). Our finding of the significant connection between motivation for interview training using an android robot and the impression of the humanness of the android robot could assist in developing the design of an android robot tailored for interview training. In addition, the present study confirmed that individuals with ASD exhibited higher motivation when interviewing with an android robot than with a human.

A previous study (22) concluded that robots designed to interact with individuals with ASD should be less detailed and less visually complex than humans, while still conforming to the humanoid form. In addition, there is a notion shared by researchers that “simpler is better,” i.e., individuals with ASD prefer simple objects (23). These factors may extend to the result of the current study that the motivation for interview training using an android robot negatively correlated with the impression of humanness. The novel finding in the present study was that among the simplicity, the “impression” of simplicity in the appearance of the robot for individuals with ASD is notable. Individuals with ASD do not observe the majority of objects in a typical manner (24); their impression of simplicity is important.

For individuals with ASD, the flood of social cues may be a primary cause for the inability to process social signals (25); it is therefore rational that ASD subjects performed better with a robot partner than with a human partner. The next interesting question pertains to which appearance of a humanoid robot is most appropriate for robotic intervention for interview training. Given that the ultimate goal of robot-assisted ASD therapy is the generalization of social skills obtained during the robot sessions to subsequent interactions with humans, an android robot is the most appropriate. We have demonstrated in the present study that individuals with ASD have higher motivation for interaction with an android robot than with a human interviewer, and the motivation was negatively related to the impression of the humanness of the android robot. The preparation of the android robot for interview training is the next issue.

The realism of a robot can be established not only by physical appearance but also by varying the levels of biological motion (26). A robot with multiple DOF vehicles has the potential to change the impression of the humanness (i.e., a robot that can move its eyes with multiple DOF appears more human-like than a robot that can merely open and close its eyes in a single plane of motion). Extensive actuation induces the perception that the robot is more realistic (26). The impression of humanness is also affected by the extent of autonomous robot behavior, such as eye, mouth, and neck movements (for example, adjusting the frequency of eye blinks) (27). Thus, adjusting the degree of actuation and the extent of autonomous action potentially increases the motivation of individuals with ASD for interview training with an android robot and may be important for such an android robot to be more effective in an interview setting.

We would like to acknowledge several limitations of our study. The first is the relatively small number of participants. Larger sample sizes are necessary to provide more meaningful data regarding the impression toward the android robot. A second limitation is the comparatively short interaction between the participants and the robot; however, 10 min per session might be appropriate for the specific characteristics of individuals with ASD, and all participants completed the trial. Third, this study was a single-session study and did not provide any indication of whether participants would respond similarly over multiple sessions. Multiple sessions may offer a more extensive understanding of habituation to the android robot over time. While the current study did not test the effects of habituation in any manner, it represents one of the first investigations of the motivation to practice an interview with an android robot and the subjects’ impression of the nature of the android robot in terms of humanness. Future studies should evaluate the effects of habituation with the robots by conducting the investigation over an extended period. Finally, only individuals with ASD were included. To more clearly elucidate the relationship between motivation and the impression of the humanness of an android robot, it is important to study individuals without ASD and compare their data to those of individuals with ASD.

The overall conclusion of this study was that motivation for interview training is negatively related to the impression of the humanness of an android robot. If we can assess the impression of the humanness objectively, by collating the results of this study, it is possible that we can control the participants’ motivation. This study is one step on the path to a complete understanding of how such an android robot should be designed and implemented to provide sufficiently realistic situations that can be of therapeutic value. This study was conducted in a single-session setting; future research to evaluate the responses in multiple sessions is needed.

## Ethics Statement

This study was carried out in accordance with the recommendations of ethics committee of Kanazawa University. The protocol was approved by the ethics committee of Kanazawa University. All subjects gave written informed consent in accordance with the Declaration of Helsinki.

## Author Contributions

HK designed the study, conducted the experiment, carried out the statistical analyses, analyzed and interpreted data, and drafted the manuscript. TM, YY, YM, MMiy, HI, MMim, YM, and MK conceived of the study, participated in its design, assisted with the data collection and scoring of behavioral measures, analyzed and interpreted the data, and were involved in drafting the manuscript and revising it critically for important intellectual content. MK was involved in providing final approval of the version to be published. All authors read and approved the final manuscript.

## Funding

This work was supported in part by Grants-in-Aid for Scientific Research from the Japan Society for the Promotion of Science (18H02746), ERATO ISHIGURO Symbiotic Human-Robot Interaction Project, and was partially supported by The Center of Innovation Program from the Japan Science and Technology Agency, JST, Japan.

## Conflict of Interest Statement

The authors declare that the research was conducted in the absence of any commercial or financial relationships that could be construed as a potential conflict of interest.
