# Supplementary material for: How the Realism of Robot Is Needed for Individuals With Autism Spectrum Disorders in an Interview Setting
Source: Front Psychiatry. 2019 Jul 11;10:486. doi: 10.3389/fpsyt.2019.00486 (PMC6637027; doi:10.3389/fpsyt.2019.00486)
Supplement: Supplementary file 1 [file DataSheet_1.docx]

**Supplementary material**

(1) Examples of the scripts

1. Please take a seat.
2. Good afternoon. Hello there.
3. Thank you for applying, my company XX.
4. Well then, could you introduce yourself?
5. Would you please tell me the reasons of your application?
6. Why would you like to get a job? Please tell me.
7. What are the things you are good at?

What is your special talent?

1. What are the things you are not good at?
2. How are you coping with the things you are weak in?
3. What kind of things would you like to do in our company?

Please tell me what you can do.

1. What are you doing in the vocational training school? From what time until what time is it?
2. Have you ever failed at your work?
3. Please explain your disability briefly.
4. How are you feeling now?
5. In my company, we have a lot of jobs that need standing. Is that all right?
6. Is it fine with you to carry heavy stuff?
7. Are you on any medicine now?
8. Is there anything you would like us to consider?
9. Which route did you take to come here from your home?
10. Are there any working days or working hours you particularly wish?
11. Is there anything else you would like to talk to us?
12. I understand. Thank you very much.
