# Supplementary material for: How the Realism of Robot Is Needed for Individuals With Autism Spectrum Disorders in an Interview Setting
Source: Front Psychiatry. 2019 Jul 11;10:486. doi: 10.3389/fpsyt.2019.00486 (PMC6637027; doi:10.3389/fpsyt.2019.00486)
Supplement: Supplementary file 2 [file DataSheet_2.pdf]

name	motivation	Humanness	emotion	animatedness	naturalness	familiarity	warmth	simplicity	regularity	Total
a	4	1	3	3	2	2	2	3	4	20
b	5	1	2	3	2	1	2	3	4	18
c	3	5	5	4	4	5	4	3	2	32
d	2	3	4	2	4	2	4	5	3	27
e	3	2	3	4	4	3	2	3	4	25
f	3	5	5	3	4	3	3	2	3	28
g	5	1	1	1	1	2	2	4	4	16
h	5	2	1	2	2	2	1	5	2	17
i	3	3	3	5	4	2	1	5	5	28
j	4	4	4	4	4	5	4	1	3	29
k	4	4	3	2	2	3	4	3	3	24
l	2	5	4	4	1	3	3	3	5	28
m	5	3	1	1	3	2	1	3	5	19
n	3	2	3	2	2	1	3	3	5	21
o	2	4	3	3	3	2	2	4	2	23
p	2	2	3	3	2	2	2	5	5	24
q	3	2	3	2	2	2	3	3	4	21
r	4	2	1	1	2	1	1	5	4	17
s	4	4	3	3	3	3	3	3	3	25
t	5	1	2	2	2	1	1	2	4	15
u	4	1	2	3	3	1	1	4	3	18
v	5	1	3	4	2	4	1	2	3	20
w	3	2	2	2	1	2	1	5	3	18
